# Deep sternal wound infection after cardiac surgery

**DOI:** 10.1186/1749-8090-8-132

**Published:** 2013-05-20

**Authors:** Hiroshi Kubota, Hiroaki Miyata, Noboru Motomura, Minoru Ono, Shinichi Takamoto, Kiyonori Harii, Norihiko Oura, Shinichi Hirabayashi, Shunei Kyo

**Affiliations:** 1 Department of Cardiovascular Surgery, Kyorin University, 6-20-2, Shinkawa, Mitaka, Tokyo, Japan; 2 Japan Cardiovascular Surgery Database Organization, 7-3-1, Hongo, Bunkyo, Tokyo, Japan; 3 Department of Cardiac Surgery, University of Tokyo, 7-3-1, Hongo, Bunkyo, Tokyo, Japan; 4 Department of Plastic Surgery, Kyorin University, 6-20-2, Shinkawa, Mitaka, Tokyo, Japan; 5 Department of Plastic Surgery, Teikyo University, 2-11-1, Kaga, Itabashi, Tokyo, Japan

**Keywords:** Deep sternal wound infection, Cardiac surgery, Outcome, Re-exploration for bleeding, Operative mortality, Risk factor

## Abstract

**Background:**

Deep sternal wound infection (DSWI) is a serious postoperative complication of cardiac surgery. In this study we investigated the incidence of DSWI and effect of re-exploration for bleeding on DSWI mortality.

**Methods:**

We reviewed 73,700 cases registered in the Japan Adult Cardiovascular Surgery Database (JACVSD) during the period from 2004 to 2009 and divided them into five groups: 26,597 of isolated coronary artery bypass graft (CABG) cases, 23,136 valvular surgery cases, 17,441 thoracic aortic surgery cases, 4,726 valvular surgery plus CABG cases, and 1,800 thoracic aortic surgery plus CABG cases. We calculated the overall incidence of postoperative DSWI, incidence of postoperative DSWI according to operative procedure, 30-day mortality and operative mortality of postoperative DSWI cases according to operative procedure, 30-day mortality and operative mortality of postoperative DSWI according to whether re-exploration for bleeding, and the intervals between the operation and deaths according to whether re-exploration for bleeding were investigated. Operative mortality is defined as in-hospital or 30-day mortality. Risk factors for DSWI were also examined.

**Results:**

The overall incidence of postoperative DSWI was 1.8%. The incidence of postoperative DSWI was 1.8% after isolated CABG, 1.3% after valve surgery, 2.8% after valve surgery plus CABG, 1.9% after thoracic aortic surgery, and 3.4% after thoracic aortic surgery plus CABG. The 30-day and operative mortality in patients with DSWI was higher after more complicated operative procedures. The incidence of re-exploration for bleeding in DSWI cases was 11.1%. The overall 30-day/operative mortality after DSWI with re-exploration for bleeding was 23.0%/48.0%, and it was significantly higher than in the absence of re-exploration for bleeding (8.1%/22.0%). The difference between the intervals between the operation and death according to whether re-exploration for bleeding had been performed was not significant. Age and cardiogenic shock were significant risk factors related to re-exploration for bleeding, and diabetes control was a significant risk factor related to DSWI for all surgical groups. Previous CABG was a significant risk factor related to both re-exploration for bleeding and DSWI for all surgical groups.

**Conclusions:**

The incidence of DSWI after cardiac surgery according to the data entered in the JACVSD registry during the period from 2004 to 2009 was 1.8%, and more complicated procedures were followed by higher incidence and mortality. When re-exploration for bleeding was performed, mortality was significantly higher than when it was not performed. Prevention of DSWI and establishment of an effective appropriate treatment for DSWI may improve the outcome of cardiac surgery.

## Background

Infection of the sternotomy wound is a serious complication of open heart surgery. It is a potentially devastating and occasionally fatal complication.

The aim of this study was to determine the overall incidence of DSWI after cardiac surgery, the incidence of DSWI according to each operative procedure, the overall 30-day mortality and operative mortality in patients with DSWI, the 30-day mortality and operative mortality in patients with DSWI according to operative procedure, the effect of re-exploration surgery due to postoperative bleeding on DSWI mortality, and risk factors related to re-exploration for bleeding and DSWI in a series of cases recorded in JACVSD.

## Methods

Because this study involved human subjects, approval was sought and obtained from the Institutional Review Board of each participating hospital. Informed consent had been obtained from each patient to allow his or her data to be entered into the JACVSD.

The JACVSD was inaugurated in 2000 to make it possible to assess outcomes after cardiovascular surgical procedures on a multicenter basis. As of 2012, it captures clinical information from 485 hospitals 2012 (82% of all units performing cardiac surgery in 2012), and we included data from the 178 hospitals that had enrolled by December 2009 in this study. The usage of database for this study was approved by the Data Utilization Committee in the JACVSD.

Of the 81,796 adult cases recorded in the JACVSD during the period from 2004 to 2009, we reviewed the 73,700 cases that did not involve miscellaneous cardiac procedures (8,096 cases) and divided them into five groups: 26,597 isolated coronary artery bypass graft (CABG) cases, 23,136 valvular surgery cases, 17,441 thoracic aortic surgery cases, 4,726 valvular surgery plus CABG cases, and 1,800 thoracic aortic surgery plus CABG cases. We investigated the following in regard to these 26,597 cases:

1. Overall incidence of postoperative DSWI

2. Incidence of postoperative DSWI according to operative procedure.

3. Overall incidence of re-exploration for bleeding in patients without/with postoperative DSWI.

4. Incidence of re-exploration for bleeding in patients without/with postoperative DSWI according to operative procedure.

5. Overall 30-day mortality and operative mortality in patients with postoperative DSWI.

6. The 30-day mortality and operative mortality of DSWI cases for each operative procedure.

7. Overall 30-day mortality and operative mortality in patients with DSWI according to whether re-exploration for postoperative bleeding.

8. The 30-day mortality and operative mortality in patients with DSWI after each operative procedure according to whether re-exploration for postoperative bleeding.

9. The intervals between the operation and death and effect of re-exploration for bleeding on mortality.

Risk models of re-exploration for bleeding and DSWI for each procedure were referred in the (Appendix).

### Statistical analysis

Operative mortality is defined as in-hospital or 30-day mortality (whichever longer), which is equivalent to “the 30-day operative mortality” as defined in the STS National Adult Cardiac Surgery Database.

Also definition of DSWI in JACVSD were “indicate whether the patient, within 30 postoperatively, had a deep sternal infection involving muscle, bone, and/or mediastinum requiring operative intervention, and have any of the following conditions: 1. Wound opened with excision of tissue (I&D) or re-exploration of mediastinum. 2. Positive culture. 3. Treatment with antibiotics.”

## Results

1. Overall incidence of postoperative DSWI was 1.8%.

2. The incidence of postoperative DSWI was 1.8% in isolated CABG group, 1.3% in valvular surgery, 2.8% in valvular surgery concomitant with CABG, 1.9% in thoracic aortic surgery, 3.4% in thoracic aortic surgery concomitant with CABG.

3. Overall incidence of re-exploration for bleeding in patients without/with postoperative DSWI was 3.6/11.1%* (*: p < 0.05).

4. The incidence of re-exploration for bleeding without/with DSWI according to operative procedure was 1.8%/6.9%* in isolated CABG group, 3.8/11.8%* in valvular surgery, 4.9/6.1% (n.s.) in valvular surgery concomitant with CABG, 5.4/15.9%* in thoracic aortic surgery, 9.1/25.8%* in thoracic aortic surgery concomitant with CABG.

5. Overall 30-day mortality and operative mortality in patients with DSWI was 9.7% and 25.8% respectively.

6. The 30-day mortality in patients and operative mortality with DSWIwere 5.2% and 19.0% in isolated CABG, 10.5% and 23.0% in valvular surgery, 10.0% and 22.3% in valvular surgery concomitant with CABG, 14.1% and 34.9% in thoracic aortic surgery, 17.7% and 50.0% in thoracic aortic surgery concomitant with CABG respectively.

7. Overall 30-day mortality and operative mortality in patients with DSWI with re-exploration for bleeding was 23.0% and 48.0% respectively. It was significantly higher than 30-day mortality in patients with DSWI without re-exploration for postoperative bleeding (8.1% and 22.0%, p < 0.05).

8. The 30-day mortality and operative mortality in patients with DSWI of each operative procedure without/with re-exploration due to postoperative bleeding was 4.3/18.2%* and 17.5/39.4%* in isolated CABG, 8.8/22.9%* and 20.7/40.0%* in valvular surgery, 10.7/0% (n.s.) and 22.1/25.0% (n.s.) in valvular surgery concomitant with CABG, 11.6/26.9%* and 30.5/57.7%* in thoracic aortic surgery, 13.0/31.3%* and 43,5/68.8% (n.s.) in thoracic aortic surgery concomitant with CABG respectively.

9. In dead cases, duration from the operation to death without/with re-exploration for bleeding was 78.1/68.2 days. According to procedurs, 93.4/63.1 days (n.s.) in isolated CABG, 55.5/62.2 days (n.s.) in valvular surgery, 62.4/41.5 days (n.s.) in valvular surgery concomitant with CABG, 77.5/67.2 days (n.s.) in thoracic aortic surgery group, 95.7/90.0 days (n.s.) in thoracic aortic surgery concomitant with CABG (n.s.), (Table 1. Result 3 and 4 were separately shown in Table 2).

**Table 1:** Deep sternal wound infection after cardiac surgery

					**CABG**	**Valve**	**Valve + CABG**	**TA**	**TA + CABG**
**Total**			**73,700 (cases)**		26,597	23,136	4,726	17,441	1,800
**DSWI**	Incidence		1.8 (%)		1.8	1.3	2.8	1.9	3.4
	30 day mortality	Total	9.7 (%)		5.2	10.5	10.0	14.1	17.7
		Re-exploration for bleeding	-	8.1 (%)	4.3	8.8	10.7	11.6	13.0
			+	23.0 (%)	18.2	22.9	0	26.9	31.3
	Operative mortality	Total	25.8 (%)		19.0	23.0	22.3	34.9	50.0
		Re-exploration for bleeding	-	22.0 (%)	17.5	20.7	22.1	30.5	43.5
			+	48.0 (%)	39.4	40.0	25.0	57.7	68.8
	Duration until death	Re-exploration for bleeding	-	78.1 (days)	93.4	55.5	62.4	77.5	95.7
			+	68.2 (days)	62.1	62.2	41.5	67.2	90.0

DSWI; *deep sternal wound infection*, CABG; *coronary artery bypass graft*, TA; *thoracic aortic surgery.*

**Table 2:** Incidence of re-exploration for bleeding in patients with postoperative DSWI

	**CABG**	**Valve**	**Valve + CABG**	**TA**	**TA + CABG**
Overall incidence of re-exploration for bleeding	1.8	3.8	4.9	5.4	9.1
3.6 (%)					
Incidence of re-exploration for bleeding in DSWI cases	6.9	11.8	6.1	15.9	25.8
11.1 (%)					

## Discussion

According to the literature, the incidence of DSWI after cardiac surgery has been variously reported as between 0.8 and 5.0% [1-7]. This postoperative complication is a serious one, being responsible for a mortality rate varies between 19% and 29% in different series of adult cardiac surgical patients [1-7]. Based on JACVSD registered data from 2004 to 2009, the overall incidence of DSWI after cardiac surgery was 1.8%, as for each operative procedure, the incidence of postoperative DSWI was 1.8% in isolated CABG group, 1.3% in valve group, 2.8% in valve with CABG group, 1.9% in thoracic aorta group and 3.4% in thoracic aorta with CABG group. When operative procedure concomitant with CABG was done, the incidence of postoperative DSWI showed 1.5% of elevation compared with isolated original valve and thoracic aortic procedure. Internal thoracic artery use, prolonged operative time and longer cardiopulmonary bypass time are possible mechanisms to explain these increased rates of DSWI.

Postoperative DSWI patients showed very high 30-day/operative mortality. Overall, it was 9.7/25.8% and as for each procedure, thoracic aortic surgery concomitant with CABG showed the highest mortality, aortic related surgery and valve related surgery followed, coronary bypass surgery showed the lowest. These results represent that additionally to the above described well-known risk factors to augment the risk of DSWI after cardiac surgery, deep hypothermia and usage of synthetic graft may worsen the result of DSWI related to aortic surgery.

These results show that prevention of postoperative DSWI and establishment of the appropriate treatment for postoperative DSWI are the important factors to reduce the mortality after cardiac surgery.

Large retrospective and prospective outcome studies have identified epidemiologic factors associated with DSWI. A partial list of commonly cited risks includes advanced age, obesity, diabetes, smoking, prolonged operative time, use of internal thoracic artery conduits, hemodyalisis, reoperative surgery and prolonged cardiopulmonary bypass time [3,7,8]. In our study, previous CABG history was the most significant risk factor related to both re-exploration for bleeding and DSWI for all surgical groups. Age and cardiogenic shock were significant risk factors related to re-exploration for bleeding for all surgical groups, male gender, emergent or salvage operation, and operation combined with CABG were significant risk factors related to re-exploration for bleeding in two of three surgical groups. Diabetes control was a significant risk factor related to DSWI for all surgical groups. BMI, renal failure, COPD, cardiogenic shock, administration of inotropic agents, and triple vessel disease were significant risk factors related to DSWI in two of three groups. Aortic rupture and aortic arch operation were significant risk factors related to both re-exploration for bleeding and DSWI in thoracic aortic surgery group.

Thirty-day/operative mortality in patients with DSWI with re-exploration for bleeding was significantly higher than DSWI without re-exploration in all operative procedure except two. Although there was no thirty-day mortality in valvular surgery concomitant with CABG group with re-exploration for bleeding, there was no significant between without re-exploration group.

It is known that patients who need re-exploration for bleeding after cardiac surgery are at higher risk of complications, morbidity and mortality. In our study, the incidence of re-exploration for bleeding in DSWI cases was 11.1% and it was threefold as many as overall incidence of re-exploration for bleeding. Patients requiring resternotomy are at greater risk from the hazards of transfusion reactions, viral infections, suppression of the immune system [9,10]. Canadyova et al. described that risk factors associated with higher in-hospital mortality after re-exploration for bleeding and tamponade include delayed resternotomy, higher levels of lactate, lower levels of hematoclit before revision, older age, more complex cardiac procedures, redo operations, longer cardiopulmonary bypass, renal failure and diabetes mellitus. If the time until re-exploration is prolonged, risk of complications, morbidity and mortality will elevate [11]. Kristensen et al. reported a case-note review on propensity-matched patients to assess the outcome of reoperation for bleeding regarding morbidity and mortality. In total, 101 cases (7.0%) among 1452 consecutive patients undergoing cardiac surgery using cardiopulmonary bypass underwent surgical re-exploration due to excessive postoperative bleeding [12]. Significant risk factors for reoperation for bleeding after cardiac surgery was low ejection fraction, high EuroSCORE, procedures other than isolated CABG, elongated time on cardiopulmonary bypass, low body mass index, diabetes mellitus and preoperatively elevated s-creatinine. Reoperated patients significantly had a greater increase in postoperative s-creatinine and higher mortality. Surviving reoperated patients significantly had a lower EuroSCORE and a shorter time on cardiopulmonary bypass compared with non-survivors. The average time to re-operation was 155 min longer for non-survivors when compared with survivors. Considering these results, first of all, to secure hemostasis to prevent re-exploration for bleeding is important to prevent DSWI, and when re-exprolation for bleeding is required, prevention and earlier decision should be made to decrease DSWI after cardiac surgery.

Deniz et al. studied the effectiveness of negative pressure wound therapy compared with conventional treatment outcomes at postoperative mediastinitis after cardiac surgery in 90 patients. Because the 90-days mortality was found significantly lower (8.5% vs. 23.2%) and overall survival at 1 year was significantly better (91.5% vs. 76.7%) in the negative pressure wound group than in the conventionally treated group, they concluded that negative pressure wound therapy was safe and reliable option in mediastinitis after cardiac surgery, with excellent survival and low failure rate when compared with conventional treatments [13].

Sachithanadan et al. assessed the impact of DSWI on in-hospital mortality and mid-term survival following cardiac surgery in 4586 consecutive adult patients who underwent cardiac surgery via a median sternotomy. DSWI requiring revision surgery developed in 1.65% patients. Age, diabetes, a smoking history and ventilation time were identified as independent predictors of a DSWI. DSWI patients were more likely to develop renal failure, require reventilation and a tracheostomy postoperatively. However, they detected that DSWI is not an independent predictor of higher in-hospital mortality or reduced mid-term survival compared with the patients without DSWI following cardiac surgery. They treated using vacuum assisted closure therapy in 81.5% patients [14].

Because VAC system was approved to commercial use in 2010 in Japan and presented data was pre-approval VAC system era, their results suggest that development of the appropriate treatment surely contribute to improve the prognosis of DWSI after cardiac surgery.

## Conclusion

The incidence of deep sternal wound infection after open heart surgery was 1.8% in JACVSD registry from 2004 to 2009. Once, deep sternum infection occurs, it showed high mortality. Especially, more invasive procedure showed higher mortality. When re-exploration due to bleeding was required, the mortality elevated extremely high. DSWI is severe complication after open heart surgery. Prevention of DSWI, avoiding to re-exploration for bleeding, earlier decision for re-exploration for bleeding, contrivance of appropriate means of wound closure, and development of the appropriate treatment for DSWI are important to improve the prognosis of cardiac surgery.

## Appendix

In addition to this analysis we also refer risk model of DSWI and re-exploration for bleeding for each procedures (Details of model development and evaluation were mentioned at http://www.jacvsd.umin.jp). Previous CABG history was a significant risk factor related to both re-exploration for bleeding and DSWI for all surgical groups. Age and cardiogenic shock were significant risk factors related to re-exploration for bleeding, and diabetes control was a significant risk factor related to DSWI for all surgical groups. Male gender, emergent or salvage operation, and operation combined with CABG were significant risk factors related to re-exploration for bleeding in two of three surgical groups. Body mass index (BMI), renal failure, chronic obstructive pulmonary disease (COPD), cardiogenic shock, administration of inotropic agents, and triple vessel disease were significant risk factors related to DSWI in two of three groups. Aortic rupture and aortic arch operation were significant risk factors related to both re-exploration for bleeding and DSWI in thoracic aortic surgery group (Table 3).

**Table 3:** Risk model for re-exploration for bleeding and deep sternum wound infection

	**Re-exploration for bleeding**			**Deep sternum wound infection**		
	**IsolatedCABG**	**Valve**	**Aorta**	**Isolated CABG**	**Valve**	**Aorta**
Age (≦60,61≦65,66≦70,71≦75,76≦80,81≦)	1.09	1.13	1.07		**1.15**	
BSA × 10	0.93	0.93				
Male gender		1.44	1.27			
BMI (30≦)					**2.19**	**2.66**
Smoking history (Yes)		━			**━**	**1.51**
Current smoker (Yes)		1.23				
Diabetes control(Yes)				**1.27**	**1.28**	**1.44**
Renal failure(Yes)	1.94			**1.77**	**1.53**	
Dialysis (Yes)					**1.54**	
Hypertension(Yes)				**1.26**		
Infectious endocarditis (Yes)	━			**━**		**2.53**
Infectious endocarditis (Active)	━		1.93	**━**		
COPD (Mild, Moderate, Severe)				**1.94**		**1.74**
Carotid artery lesion (Yes(unilateral, bilateral))	━			**━**	**1.69**	
Extracardiac arteriopathy (Yes(Thoracic aorta, Peripheral artery including abdominal aorta))		1.37	1.28			
Extracardiac arteriopathy (Peripheral artery including abdominal aorta)			━	**1.46**		**━**
Consciousness Disorder within 24 hours (Yes)		1.59			**2.49**	
Previous surgery (CABG)(Yes)	5.17	2.25	2.13	**2.06**	**1.69**	**1.76**
Congestive heart failure (Yes)		1.37		**1.45**		
Cardiogenic shock (Yes)	1.46	1.66	1.43	**1.65**	**1.80**	
Arrhythmia (Yes)					**1.28**	
NYHA(II and over)	━		1.79	**━**		
Inotropic Agents(Yes)					**1.83**	**1.80**
Mitral Stenosis (Yes)		1.28	━			**━**
3 vessel disease	1.29			**1.52**	**1.59**	
2 or more vessel disease	━	━		**━**	**━**	**1.95**
LV Function(Medium)			1.16			
LV Function(bad)	1.71					
Aortic Insufficiency(IIand over)			1.24			
Mitral Insufficiency (IIand over)	━			**━**	**1.22**	
Tricuspid Insufficiency (IIand over)	━	1.29		**━**		
Operation status (Emergent, Salvage)	1.75		1.43			
Operation status (Urgent, Emergent, Salvage)	━			**━**		**1.78**
CABG (Yes)	━	1.24	1.81	**━**	**1.59**	
Unplanned CABG(Yes)						
Aortic valve surgery (Yes)		1.32				**1.39**
Aortic valve surgery (Replacement)	━		1.59	**━**		
Combined valve surgery	━	1.21	━	**━**		**━**
Aortic Aneurysm Type(Dissection)	━	━	1.38	**━**	**━**	
Aortic Aneurysm Type(Pseudoaneurysm)	━	━		**━**	**━**	**2.09**
Cause of operation(Rupture)	━	━	1.62	**━**	**━**	**1.40**
Operation site (Arch)	━	━	1.4	**━**	**━**	**2.12**
Operation site (Root)	━	━		**━**	**━**	**1.45**
Operation site (Ascending)	━	━		**━**	**━**	**1.47**
Operation site (Thoracoabdominal)	━	━	1.65	**━**	**━**	
Maze operation (Yes)	━	━	1.88	**━**	**━**	

━; not included in questionnaire, *BSA; body surface area,* BMI; *body mass index,* COPD; *chronic obstructive pulmonary disease,* CVA; *cerebrovascular accident,* TIA; *transient ischemic attack,* RIND; *reversible ischemic neurological deficit,* CCS; *Canadian cardiovascular society classification,* NYHA; *New York heart association,* LV; *left ventricle.*

## Abbreviations

DSWI: Deep sternal wound infection; JACVSD: Japan adult cardiovascular surgery database; CABG: Coronary artery bypass graft.

## Competing interests

The authors declare that they have no competing interests.

## Authors’ contributions

HK is the primary author of the manuscript. HM, NM, MO, ST, and SK have made substantial contributions to conception and design, and analysis and interpretation of the data. KH, NO, SH have revised the manuscript for important intellectual content. All authors read and approved the final manuscript.
